# Medical Society of the State of Pennsylvania

**Published:** 1860-07

**Authors:** 


MEDICAL SOCIETY OF THE STATE OF PENNSYLVANIA.—The twelfth annual session of this body was held in this city, on the thirteenth of June, Dr. Condie, President, in the chair. The delegates, nearly one hundred in number, were welcomed to the city, in a brief address, by Dr. S. D. Gross, and the annual address by the retiring president was delivered, embracing, among other subjects, the importance of the operation of vaccination, and the necessity of preserving the purity of the vaccine virus. Among the interesting subjects presented on the first day, the following important preamble and resolutions, offered by Dr. Henry Hartshorne, were unanimously adopted:—

*Whereas*, experience has shown that inebriety is in many instances a disease; the morbid craving for stimulants beyond control of the individual so long as opportunity for its indulgence exists; and whereas, for the suitable treatment and cure of this disease, accommodations are not to be found in any of our lunatic asylums or general hospitals; therefore,

*Resolved*, That in the opinion of this Society, the establishment of an Asylum for Inebriates in the State of Pennsylvania is highly desirable and important.

*Resolved*, That such modifications of existing laws as are necessary to make such an institution available by placing the persons of habitual inebriates under due limitations and control of proper authorities, are desirable, and should be urged upon our Legislature.

Reports of the Indiana, Perry, and Bradford County Medical Societies were read, and referred to the Committee on Publication, and Dr. Kennedy presented a report on Meteorology. Dr. Albert Day, Superintendent of the Washington Home, in Boston, addressed the Society on the subject of reformation of inebriates.

On the second day of the meeting, Dr. Gross read a paper on *Prostatorrhœa*, which will be found among our original contributions. Dr. Bell offered a long preamble, showing the necessity of improving the medical education of youth; and, after some discussion, resolutions relative to the best mode of enabling young students to acquire a fair knowledge of the different departments of Medicine, Surgery, and the accessory branches, were adopted. Resolutions were offered in relation to withholding all support from the professors and graduates of Female Medical Colleges, and it was settled that it would be improper, under the existing code of medical ethics, for any member of the Society to consult with female doctors.

On the third day a committee was appointed to petition the Legislature for the passage of a law more stringent in regard to the subject of criminal abortion; and the following gentlemen were elected officers for the ensuing year:—*President*, Dr. Edward Wallace, of Berks County; *Vice-Presidents*, Dr. John Bell, of Philadelphia, Dr. G. W. Allison, of Beaver, Dr. J. H. Landis, of Blair, and Dr. G. W. Lott, of Columbia County; *Corresponding Secretary*, Dr. James Carson, of Philadelphia; *Recording Secretaries*, Dr. J. T. Carpenter, of Schuylkill, and Dr. J. H. Smaltz, of Philadelphia. The Censors, Committee of Publication, and Delegates to the American Medical Association, having been appointed, the President elect was led to the chair, and made a brief address.

The Society then adjourned to meet at Pittsburg, on the second Wednesday in June, 1861. During the session of the Society its members were entertained by Dr. Atlee, and by Dr. Kirkbride, at the Pennsylvania Hospital for the Insane, and by Dr. Worthington, at the Frankford Insane Asylum.

